# Duration of Maternal Antibodies against Canine Distemper Virus and Hendra Virus in Pteropid Bats

**DOI:** 10.1371/journal.pone.0067584

**Published:** 2013-06-27

**Authors:** Jonathan H. Epstein, Michelle L. Baker, Carlos Zambrana-Torrelio, Deborah Middleton, Jennifer A. Barr, Edward DuBovi, Victoria Boyd, Brian Pope, Shawn Todd, Gary Crameri, Allyson Walsh, Katey Pelican, Mark D. Fielder, Angela J. Davies, Lin-Fa Wang, Peter Daszak

**Affiliations:** 1 EcoHealth Alliance, New York, New York, United States of America; 2 Commonwealth Science and Industrial Research Organization Australian Animal Health Laboratory, Geelong, Victoria, Australia; 3 Animal Health Diagnostic Center at Cornell University, Ithaca, New York, United States of America; 4 Lubee Bat Conservancy, Gainesville, Florida, United States of America; 5 San Diego Zoo Institute for Conservation Research, Escondido, California, United States of America; 6 Department of Veterinary Population Medicine, University of Minnesota, St. Paul, Minnesota, United States of America; 7 Faculty of Science, Engineering and Computing, Kingston University, Kingston-Upon-Thames, United Kingdom; 8 Program in Emerging Infectious Diseases, Duke-NUS Graduate Medical School, Singapore; University of Pretoria, South Africa

## Abstract

Old World frugivorous bats have been identified as natural hosts for emerging zoonotic viruses of significant public health concern, including henipaviruses (Nipah and Hendra virus), Ebola virus, and Marburg virus. Epidemiological studies of these viruses in bats often utilize serology to describe viral dynamics, with particular attention paid to juveniles, whose birth increases the overall susceptibility of the population to a viral outbreak once maternal immunity wanes. However, little is understood about bat immunology, including the duration of maternal antibodies in neonates. Understanding duration of maternally derived immunity is critical for characterizing viral dynamics in bat populations, which may help assess the risk of spillover to humans. We conducted two separate studies of pregnant *Pteropus* bat species and their offspring to measure the half-life and duration of antibodies to 1) canine distemper virus antigen in vaccinated captive *Pteropus hypomelanus*; and 2) Hendra virus in wild-caught, naturally infected *Pteropus alecto*. Both of these pteropid bat species are known reservoirs for henipaviruses. We found that in both species, antibodies were transferred from dam to pup. In *P. hypomelanus* pups, titers against CDV waned over a mean period of 228.6 days (95% CI: 185.4–271.8) and had a mean terminal phase half-life of 96.0 days (CI 95%: 30.7–299.7). In *P. alecto* pups, antibodies waned over 255.13 days (95% CI: 221.0–289.3) and had a mean terminal phase half-life of 52.24 days (CI 95%: 33.76–80.83). Each species showed a duration of transferred maternal immunity of between 7.5 and 8.5 months, which was longer than has been previously estimated. These data will allow for more accurate interpretation of age-related Henipavirus serological data collected from wild pteropid bats.

## Introduction

Old world frugivorous bats of the genus *Pteropus* (family *Pteropodidae*) are reservoirs for important zoonotic paramyxoviruses, including Nipah virus and Hendra virus – both members of the genus *Henipavirus* (family *Paramyxoviridae*). Both Hendra and Nipah viruses have been associated with severe neurological and respiratory disease and high mortality rates in domestic animals and humans [1]. Hendra virus is enzootic in pteropid bats in Australia, while Nipah and Nipah-like viruses have been identified in *Pteropus* species throughout Asia and in other related pteropodid bat species in Africa [2]–[12]. Field and laboratory studies have been conducted to elucidate the viral dynamics in pteropid bats in order to better understand the timing and nature of spillover to humans. Henipaviruses appear to have an acute shedding period in bats. Experimental and natural infections in pteropid bats have resulted in viral RNA detection in excreta up to 17 days post infection and isolation within 3 weeks of apparent infection respectively, making detection of infected individuals in the wild challenging [2], [13]–[15]. As a result, field studies have largely relied on serological data to identify infection rates in free ranging bat populations. Serological studies of Nipah and Hendra virus antibodies in free-ranging pteropid bat colonies have found seroprevalence to be as high as 59% [4], [16]–[18]. However, viral isolation and molecular studies suggest a very low (<1%) incidence of infection [17], [19].

Serum neutralization tests (SNTs) are considered the gold standard for detecting specific antibodies to Hendra and Nipah virus [20]. However, the use of SNTs have been limited, particularly in countries where henipaviruses are enzootic, because they are classified as select agents and require the highest level of biocontainment (Biosafety level (BSL) 4) in order to work with the live viral cultures required to conduct neutralization assays. As BSL 4 labs are not available in most countries where henipaviruses occur, IgG Enzyme-linked immunosorbant assays (ELISAs) and Luminex assays [21] have been used to test sera for anti-Nipah or anti-Hendra antibodies because they can be performed under standard biosafety conditions [4], [22].

Using serological studies to understand the dynamics of infectious agents in wildlife presents challenges. Few serological assays have been validated for wildlife species. Further, antibodies may cross react or cross-neutralize related viral antigens, which can limit the specificity of assays. There is also very little information available about maternal transfer of immunity in pteropid bats, including how long specific antibodies remain in the pup’s blood. This makes it difficult, in studies of wild bats, to determine precisely when an animal was infected or whether a subadult may still have residual maternal immunity.

Bats, in general, undergo hemochorial placentation; have a similar repertoire of immunoglobulin subclasses (IgA, IgE, IgG and IgM) to other placental mammals; and they likely transfer maternal antibodies *in utero* like humans and non-human primates [23]–[25]. In addition, bats have been found to have a higher genetic diversity of variable heavy chain gene regions in their antibody repertoire compared to other mammals [26], [27]. Transmission of maternal immunity from mother to offspring occurs either across the placenta or the mammary gland. Little is known, in general, about *Pteropus* immunology. The structure of gamma immunoglobulin (IgG) in pteropodid bats appears to be consistent with other eutherian mammals [25]. The transfer of maternal antibodies has been observed in captive pteropid bats [15], [17], though the primary mechanism has not been described. In pteropid bat species that have been examined to date, the placenta has a hemodichorial structure, similar to that of humans and rabbits [28]. This type of placentation participates in the active transfer of IgG *in utero* [29]. Detection of antibodies to Menangle virus (MenV) in fetuses from *Pteropus poliocephalus* dams seropositive to MenV supports the transplacental transfer of maternal antibody in pteropid bats [30]. *Pteropus alecto* bats have a high abundance of IgG in their milk, a feature generally associated with species that transfer maternal immunity via colostrum to their offspring [31], [32]. Thus, it is possible that bats are capable of transferring IgG both transplacentally and across the mammary gland.

Differences in the kinetics of antibody responses have been reported in some bats, compared to conventional laboratory animals (reviewed in [33]). Antibodies appear to play a role in viral immunity, as observed in bats vaccinated against rabies virus compared to unvaccinated animals that succumb to disease [34]. The role of IgG antibodies in henipavirus infection in *Pteropus* spp. is less certain, as infected bats may not have a measurable titer and infected bats may shed virus despite having a measurable titer [2], [13]. Hendra virus antibodies have been found in *Pteropus conspicillatus* pups two-six weeks old and born to seropositive dams, and titers were proportionate to that of the dam [17]. Age-stratified analyses of juvenile seroprevalence may help elucidate viral incidence, provided the animal’s age can be accurately assessed. A study of captive *Pteropus vampyrus* measured maternal antibodies up to 14 months post-parturition [15]. An age-stratified survey of Hendra virus in Little Red flying foxes (*Pteropus scapulatus*) in Australia found that adult bats had an HeV seroprevalence of approximately 20%, while pre-weaned individuals had a 56% seroprevalence (n = 790) [35]. While juvenile seroprevalence could indicate recent viral circulation within a bat colony, the presence of maternal IgG may confound sero-epidemiological studies. Interpreting serological data from juvenile bats is challenging because the duration of maternal IgG in pups is unknown, and it is difficult to accurately determine the age of a juvenile pteropid bat that is independent from its dam but not yet sexually mature (∼6–24 months, depending on species)[36]–[38]. While the duration of maternal antibodies has been described in humans and domestic animals [39]–[44], there is little data available on the half-life or duration of maternal antibodies in pteropid bats. A longitudinal serological study of Hendra virus antibodies in *P. scapulatus* measured a decrease of antibodies in wild caught juveniles after 6 months [35]. Due to the biosafety requirements for henipaviruses and the associated costs of running experiments under BSL 4 conditions, few experimental infections have been conducted in bats, and none have been conducted that have measured the duration of maternal antibodies to henipaviruses in pups.

Here we describe two complementary studies designed to determine the duration and half-life of maternal antibodies in key bat reservoir species for henipaviruses: *P. hypomelanus*, a reservoir for Nipah virus and *P. alecto*, a primary reservoir for Hendra virus [3], [5]. In the first experiment, we vaccinated members of a captive breeding population of *P. hypomelanus* using a viral antigen closely related to Nipah virus that would allow us to measure the duration of maternal antibodies in their offspring under BSL 2 conditions. In the second experiment, we measured the duration of maternal antibodies to Hendra virus in pups born to naturally infected dams in a captive colony of *P. alecto.*

## Materials and Methods

### Experiment 1: Duration of Maternal Antibodies Against Canine Distemper Virus in Experimentally Vaccinated Pteropus Hypomelanus

This work was conducted at the Lubee Bat Conservancy in Gainesville Florida between 2007 and 2009 under IACUC CP07-1 Epstein. Twenty adult female *Pteropus hypomelanus* were introduced to two males under captive breeding conditions. After a period of 2 weeks, the males were removed from the enclosure. The females were checked for pregnancy every month using ultrasound. None of the bats in this experiment had been previously exposed to or vaccinated against canine distemper virus. All bats had also tested negative for IgG antibodies against Nipah virus at the Centers for Disease Control and Prevention (unpublished data). Pregnant females were identified within 2 months of mating and separated into a cohort. Five pregnant bats were vaccinated against canine distemper virus using a canarypox-vectored canine distemper virus vaccine (Meriel, USA) according to manufacturer’s instructions for dogs. We had previously demonstrated that this dosing regimen elicits an immune response in *P. hypomelanus* (unpublished data). Nine bats from the study group (5 pregnant and 4 non-pregnant) and 5 control group bats (non-mated) were given (1.0 ml) vaccine subcutaneously at days 0, 21, and 42 beginning at the third month of gestation. 1.0 ml of blood was drawn from the bats prior to day 0 to establish a negative titer, then blood was drawn on days 0, 3, 7, 28, 42 and 49 after initial dose and then every 30 days until the end of the study.

A blood sample (1.0 ml) was collected from either the radial artery/vein or saphenous vein using a 25 or 27 g ¾” needle for adults and a 1 ml tuberculin syringe for juveniles born to vaccinated mothers every 30 days for 24 months or until negative titers were obtained. Sampling began when the pups were a minimum weight of 100 g, at approximately 4–6 weeks of age. Sampling continued until we received titers at or below 16 at which point we considered the titer to be “negative.” In dogs, a protective titer is considered to be above 1∶32 by the Cornell Veterinary Diagnostic Lab [45], however, to date there have been no experimental challenges with CDV in bat species. The lab did not heat-treat the plasma samples according to standard practice, which allows active complement to non-specifically neutralize virus at dilutions of 1∶16 and below. Therefore, we considered a titer of 16 to be the negative cutoff in this study.

Blood was placed in an EDTA tube (vaccutainer, BD USA) and 0.5 ml plasma was sent to the Animal Health Diagnostic Center at Cornell University (Ithaca, NY) to measure the neutralizing antibodies against CDV using a neutralization test. Briefly, 50 µl of diluted plasma per well was added to 2 wells of a microtiter plate containing an equal volume of test medium. Serial 2-fold dilutions were done to the end of the plate. An equal volume of CDV (Onderstepoort strain) containing 30–100 TCID_50_ of virus was added to each well. Plates were incubated for at least 1 hr at 37°C. Then Vero cells were added in suspension to each well (∼20,000 cells per well). Plates were incubated for five days. Each well was examined for presence of typical CDV cytopathology. Wells were scored as positive or negative. Titer of plasma is the reciprocal of the dilution calculated as a 50% end point.

### Experiment 2: Duration of Maternal Antibodies Against Hendra Virus in Offspring of Naturally Infected Pteropus Alecto

This work was conducted at the CSIRO Australian Animal Health Laboratory (AAHL) in Geelong, Victoria and all work was approved by the AAHL animal ethics committee (protocols AEC1474 and AEC1532). *Pteropus alecto* were captured in the environs of Brisbane, Queensland using mist nets [17]. Bats were caught and held under Queensland EPA Scientific permit #WISP06386409 and Victorian Dept. of Primary Industries (DPI)Scientific permit #13909659; bats were imported to Victoria from Queensland under Victorian DPI Import permit #13894504. Thirteen adult female bats that were determined to be pregnant by abdominal palpation were brought into captivity at AAHL in August 2011. All thirteen bats gave birth to a single pup each between late October and early November 2011. Urine and oropharyngeal swab specimens from all adult and neonatal bats used in this experiment were screened and were negative for Hendra virus RNA using RT PCR (data not shown).

Adult females were allowed to acclimatize for a period of one week following transfer into captivity before samples were obtained and pups were sampled from one month post-partum. Blood was collected from adults and pups every 30 days until 12 months post-partum. Each adult animal was anaesthetized using isoflurane, a gas anesthetic, and 2 ml of blood was obtained from the cephalic vein using a 25 g needle. Pups were bled from the cephalic vein using a 25 g needle attached to a Multivette tube (Sarstedt) and 0.5–1 ml of blood was obtained monthly from one month to 12 months post-partum. Blood was placed in serum collection tubes (vaccutainer, BD USA). All sera were heat inactivated at 56°C for 30 minutes prior to use. Sera were tested for antibody binding to recombinant soluble Hendra virus G glycoprotein (sG_HEV_) using a Luminex multiplexed binding assay as described previously [21]. Briefly, sG_HEV_ coupled microspheres (Bio-Rad Laboratories, Inc) were incubated with sera (1∶250), followed by incubation with biotynylated Protein A/G (1∶500) (Pierce, Rockford, IL, USA), followed by streptavidin-phycoerythrin (1∶1000) (Qiagen, Doncaster, Vic, Australia). Antibodies bound to sG_HEV_ coated beads were quantified by fluorescence and read as the median fluorescence intensity (MFI) on a Bio-Plex Protein Array System integrated with Bio-Plex Manager Software (Bio-Rad Laboratories, Inc., CA, USA). MFI values <200 were considered negative [7].

In order to confirm Luminex results, serum neutralization tests (SNTs) were performed on a subset of samples from pups 204 g and 677 g and for selected time points for four adults as described previously using a starting serum dilution of 1∶20. Serum neutralization was determined by the presence of cytopathic effects (CPE) in the cellular monolayer and recorded as the serum dilution where no CPE was evident [21]. All experiments utilizing live virus were performed under BSL 4 conditions.

### Half-life Calculation

Antibody decay rates were calculated using a two-compartmental model, based on [42]. The initial (distributive) phase describes the equilibration of a biologic agent between the intra- and extravascular spaces. The terminal phase or elimination represents the actual use of the material [42], [43]. Both phases can be described by fitting independent linear models. This method also allows the determination of confidence intervals and the assessment of the model adequacy (e.g., if indeed there are two phases). Where bi-phasic decay rates were detected, the terminal half-life was used to represent the rate of antibody decay. Half-lives were estimated fitting a non-parametric regression. These analyses were performed using the statistical software R [44] and the package PK [45]. Mean duration of immunity for each group was compared using an unpaired student’s t-test [physics.csbsju.edu/cgi-bin/stats/t-test]. Pups’ titers were compared using Welch’s t test in the statistical software R.

## Results

### Experiment 1

**Figure 1:**
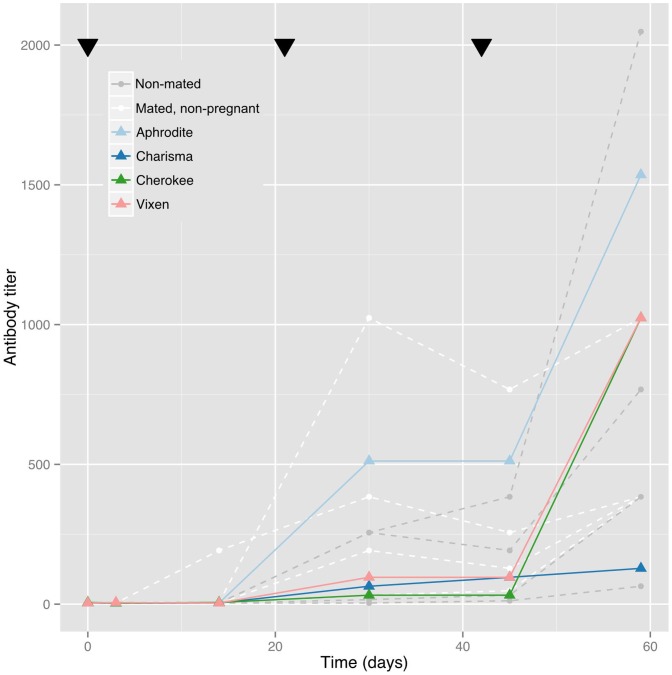
Antibody response to inoculation with a canarypox vectored canine distemper virus vaccine in adult *Pteropus hypomelanus* including those who were non-mated (control group); mated, non-pregnant; and mated, pregnant (colored lines). Black triangles indicate the administration of the three doses of vaccine on days 0, 21, and 42. Named bats in the figure are the dam of the pups described in figure 2.

**Figure 2:**
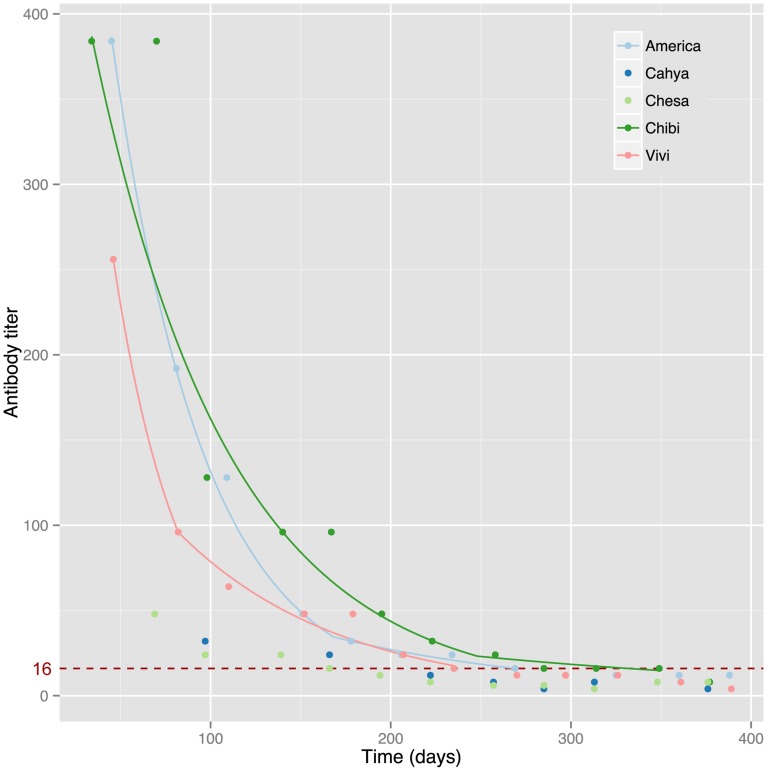
Maternal antibody titers against canine distemper virus in five neonate *Pteropus hypomelanus* beginning at 1 month post-parturition. The red dashed line indicates a negative titer cutoff of 16.

All adult bats showed an immune response to the canine distemper vaccine (**Figure 1**). Five *P. hypomelanus* pups were born to vaccinated dams. The initial and terminal half-lives, which represent the first and second phase of bi-exponential antibody decay, were calculated for each pup and are presented in **Table 1**. Two pups could not be used in the half-life calculation as they dropped below the negative cutoff in fewer than four titer measurements – the minimum required for the half-life calculation. These two pups, Cahya and Chesa, were twins belonging to Charisma, and their starting titers (mean = 40, n = 2) were significantly lower than the other three pups’ (mean = 206.5, Welch's t = 6.94, df = 2.138, p = 0.017). The three remaining pups showed bi-phasic CDV antibody half-lives. Titer curves for the pups are presented in **Figure 2**. The geometric mean (GM) of the initial half-life is 36.19 days (CI 95%: 14.6–89. 6); the GM of the Terminal half-life is 96.0 days (CI 95%: 30.7–299.7). The mean duration of immunity (titer above 16; n = 5) was 228.6 days (95% CI: 185.4–271.8) ∼7.6 months.

**Table 1:** Initial and terminal half-lives (in days) for *P. hypomelanus.*

Bat Pup Name	Initial t_1/2_ (days)	Terminal t_1/2_ (days)
America	35.33	91.00
Chibi	52.72	155.56
Vivi	25.44	62.50

Pups displayed bi-phasic rates of antibody decay. These calculations exclude pups Cahya and Chesa because there were too few observations to calculate the half-life.

### Experiment 2

**Figure 3:**
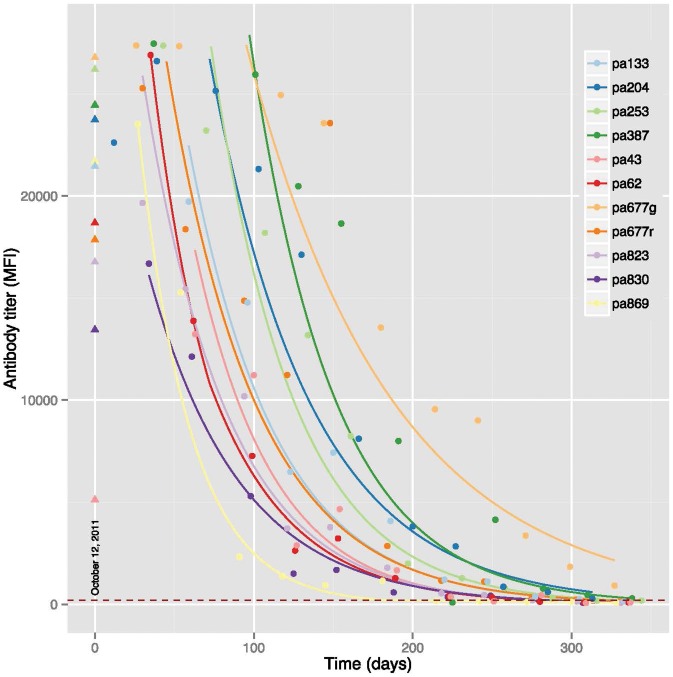
Anti-HeV titers in thirteen juvenile *P. alecto* from one month to 12 months post-partum. The anti-HeV titers of the dams are shown as triangle with corresponding colors from the measurement immediately preceding birth. Initial juvenile titers were commensurate with that of their dam. Titers are shown as median fluorescence intensities (MFI).

Initial anti-Hendra virus antibody titers from the 13 adult *P. alecto* and titers immediately preceding parturition are shown in **Figure 3**. The serum antibody titer in samples collected close to the birth of the pups displayed similar levels of HeVsG antibody to those detected following capture. Twelve pups born to seropositive dams had serum antibody to recombinant HeV sG and one pup (pup 483) born to a seronegative dam was also seronegative (data not shown). The titer curves of the pups from one to twelve months post-partum are also presented in **Figure 3**. For comparative purposes, SNTs were performed on pups pa204 and pa677 g demonstrating the presence of neutralizing antibody to HeV (data not shown). The initial and terminal half-life for each of the twelve seropositive pups and is shown in **Table 2**. Of the twelve pups, only two appeared to have biphasic half-lives, whereas 10 of 12 had initial half-lives equal to the terminal half-lives, indicating a mono-phasic decay rate. The geometric mean for the initial phase was 36.83 days (CI 95%: 29.82–45.48). The geometric mean for the terminal phase was 52.24 days (CI 95%: 33.76–80.83). The mean duration of immunity (n = 8 bats) was 255.13 days (95% CI: 221.0–289.3) ∼8.5 months. Four of the 12 pups in the study did not reach the negative cutoff within the timeframe of the study, and were omitted from the calculation of mean duration of immunity. There was no significant difference between the mean duration of immunity for Experiment 1 and 2 (t = −1.06; p = 0.3).

**Table 2:** Initial and terminal half-lives (t_ ½_) for serum antibody to sGHeV in *P. alecto* pups.

Pup ID	Initial T ½ (days)	Terminal T ½ (days)
pa677r	39.00	39.00
pa823	36.98	36.98
pa830	31.82	82.06
pa869	19.17	245.35*
pa923	39.19	39.19
pa677 g	63.17	63.17
pa387	39.92	39.92
pa253	37.12	37.12
pa204	43.99	43.99
pa133	37.87	37.87
pa62	34.24	34.24
pa43	38.98	38.98
Geometric mean		52.24 (+/−28.59)

* Pup 869 (fWanda Markotterailed to thrive and was euthanized) and Pup 483 (seronegative) were removed from the table.

## Discussion

Bats have become the subject of an increasing number of field-based epidemiological studies due to their association with zoonotic pathogens such as Ebola virus, Marburg virus, Nipah virus, Hendra virus, SARS coronavirus, and most recently a novel bat coronavirus in the Middle East– all of which cause mortality in humans [46], [47]. It has been suggested that outbreaks of viruses within bat populations have been related to the waning of immunity in juvenile cohorts. Understanding the duration of maternal antibodies in pteropid bats (and the age of the bat) will help determine whether anti-henipavirus IgG in juveniles is maternally-derived rather than the result of viral exposure. Each of the two experiments presented here provide valuable data related to bat immunology, however, there were limitations to interpreting observed differences or similarities between the results from the two experiments since they each involved different bat species and different methodologies. Experiment 1 used a canarypox vectored canine distemper virus vaccine as a proxy for Nipah virus infection, which although it was the safest option, may not have generated the same results had we been able to follow pups born to *P. hypomelanus* dams naturally infected with NiV (as with HeV in Experiment 2). Since the completion of Experiment 1, a canarypox vectored Hendra virus vaccine has been developed, and this may serve as a better surrogate for future studies requiring lower biosafety conditions [48]. We expect that the results from Experiment 2, which was based on a natural infection of a pteropid bat with Hendra virus, are more likely to be comparable to immune dynamics in closely related species infected with Nipah virus compared to those from Experiment 1, though both studies provided a controlled opportunity to measure immune system dynamics in key *Henipavirus* reservoir species.

Previous age-stratified serological studies of henipaviruses in pteropid bats have found that the sero-status of neonates matches their dam [15], [17], [35], [49]. Plowright et al., [50] described the annual occurrence of HeV spillover events in Australia as coinciding with the presence of a susceptible juvenile population of bats and estimated that maternal antibody had waned by approximately 6 months post-partum, coinciding with annual HeV spillover events. Similarly, distinct pulses of Marburg virus transmission in juvenile *Rousettus aegyptiacus* fruit bats at approximately 6 months post-partum have been reported [51]. A wave of virus infection has also been detected in *Myotis myotis* bats approximately one month after parturition which the authors speculated may be associated with waning maternal antibody [52]. A study of Nipah virus in captive *P. vampyrus* found maternal IgG to last approximately 14 months; however the exact age of the pups in that study was uncertain, and two of the four died before titers became negative [15]. We found that the calculated half-life values of maternally derived antibody did not differ significantly between pups from vaccinated bats (Experiment 1) and naturally infected bats (Experiment 2). In experiment 2, four pups whose titers did not fall below the negative threshold during the period of measurement were omitted from calculation. However, if they had an endpoint and were included, the mean duration of immunity would have been lengthened, although it cannot be determined if this would have created a statistically significant difference from Experiment 1.

Bats in both experiments showed a similar duration of maternal antibodies between 7.5 and 8.5 months in Experiments 1 and 2 respectively. Duration of maternal immunity is influenced by multiple factors including the magnitude of the mother’s titer during gestation (which can be affected by vaccination vs. natural infection), the age of the neonate at parturition (premature offspring tend to receive fewer antibodies) as well as antibody decay rate in neonates. The duration of maternal antibodies to measles virus in human infants has been shown to be longer in those born to naturally infected mothers versus mothers who were vaccinated [42]. The timeframes we observed are longer than the suggested six months estimated at the population level for Hendra virus in *P. scapulatus* [35]. However, our data from Experiment 2 do show a significant decrease in titer by six months, which represents approximately 3.5 terminal half-life periods for Hendra virus antibodies, or a decay to less than 1/8 of the starting titer, which may result in sufficiently decreased herd immunity at the population level to allow for viral circulation among the juvenile cohort.

A direct correlation was observed between the seropositivity of dams and their pups, with antibodies against CDV being detected in all five pups born to vaccinated dams, and anti-HeV antibodies detected in the 12 pups born to 12 seropositive dams and no HeV antibodies in the one pup born to a seronegative dam. This result is consistent with an earlier Hendra virus study demonstrating a strong association between dam and pup serostatus [17]. It appears from the data that the pups’ titers correlate with their dam’s. Interestingly, we found that Charisma, who had the lowest titer of the dams, produced twins (Chesa and Cayha) that had significantly lower titers than their peers. We hypothesize that there was correlation between the titers of dams and their pups, however, in this case we did not have enough data to test this statistically as the titers of Chesa and Cahya were not independent of each other and would likely skew the correlation coefficient towards significance.

We did not measure the decay rates of antibodies in adult bats, however, we would expect them to be slower than that observed in pups. Faster decay of maternally derived antibodies has been reported in human infants born to vaccinated mothers compared with naturally immune mothers [43], [53].

Henipaviruses are an important group of zoonotic viruses carried by *Pteropus* species, and understanding pteropid immunology is important for modeling the dynamics of viral infections within flying fox populations. Waning immunity to henipaviruses in juvenile cohorts may be critical to the timing of outbreaks within colonies, and therefore related to risk of spillover to humans and other animals. Further study of bat immunology will be helpful both for ecological studies of viral pathogens and also for understanding how bats respond to viral infections.
